# Supplementary material for: Hygroscopic holey graphene aerogel fibers enable highly efficient moisture capture, heat allocation and microwave absorption
Source: Nat Commun. 2022 Mar 9;13:1227. doi: 10.1038/s41467-022-28906-4 (PMC8907192; doi:10.1038/s41467-022-28906-4)
Supplement: Supplementary file 1 — Supplementary information [file 41467_2022_28906_MOESM1_ESM.pdf]

Supplementary Information

Hygroscopic holey graphene aerogel fibers enable highly efficient moisture capture, heat allocation and microwave absorption

Yinglai Hou[1,2 †], Zhizhi Sheng[2 †], Chen Fu[2], Jie Kong[1*], and Xuetong Zhang[2, 3*]

[1]Shaanxi Key Laboratory of Macromolecular Science and Technology, School of Chemistry and Chemical Engineering, Northwestern Polytechnical University, Xi'an 710072, P. R. China

[2]Suzhou Institute of Nano-Tech and Nano-Bionics, Chinese Academy of Sciences, Suzhou 215123, P. R. China

[3]Division of Surgery & Interventional Science, University College London, London NW3 2PF, U.K.

[†] These authors contributed equally: Yinglai Hou, Zhizhi Sheng.

email: kongjie@nwpu.edu.cn; xtzhang2013@sinano.ac.cn

Table of Contents

Supplementary Note 1. Materials. 3

Supplementary Note 2. Calculation details for Heat Allocation Performance............. 3

Supplementary Note 3. Measurement of Complex Permittivity. 5

Supplementary Note 4. Calculation details for Electromagnetic Absorption Performance from Complex Permittivity 5

Supplementary Note 5. Debye Relaxation... 6

Supplementary Figures 7

Supplementary Tables 27

Supplementary References 29

Supplementary Note 1. Materials.

Natural graphite powder (400 mesh), phosphorus pentoxide (P_2O_5), potassium permanganate ($KMnO_4$), hydrogen peroxide (H_2O_2, 30% aqueous solution), sulfuric acid (H_2SO_4), hydroiodic acid, lithium chloride

Supplementary Note 2. Calculation details for Heat Allocation Performance

We contracted a characteristic curve to make the loading dependent on only one free variable. The used variable is the sorption potential (A), which is the Gibbs free energy of the sorption process:[1-2]

$$A = -RT\ln(\frac{p}{p_s}) \quad \textbf{(1)}$$

Where p_s is the saturated vapor pressure, p is the pressure at each loading value, and T is the analyse temperature. In order to prove the validity of the characteristic curve model, the calculated curve at different temperatures should be coincident.

The isosteric enthalpy of sorption for water, which can be calculated using the Clausius-Clapeyron equation: [2]

$$\Delta H_{ads} = -Rln(\frac{p_2}{p_1})\frac{T_1 \times T_2}{T_2 - T_1} \quad \textbf{(2)}$$

Where, ΔH_{ads} is the sorption enthalpy of water on the sorbent and R refers to the ideal gas constant (8.314 J·mol^{-1}·K^{-1}), p_1 and p_2 are corresponding vapor pressure when the specific sorption capacity is equal at T_1 and T_2, respectively.

Q_{eva} is the energy taken up by evaporation, Q_{con} is the energy released by the condenser, Q_{ads} is the energy gained during the adsorption process, and Q_{regen} is the energy required by regeneration, they can be calculated by[2-4]

$$Q_{eva} = \Delta H_{eva}(T_{eva})\Delta w \quad \textbf{(3)}$$

$$Q_{cond} = \Delta H_{con}(T_{con})\Delta w \quad \textbf{(4)}$$

Where ΔH_{eva} is evaporation enthalpy and ΔH_{con} is the condensation enthalpy. T_{eva} and T_{con} are temperatures of evaporator and condenser, respectively.

$$Q_{ads} = \int_{T_{des}}^{T_{IC}} C_p^{sorbent}(T)dT + \int_{T_{des}}^{T_{IC}} \rho_{liq}^{wf} w_{max} C_p^{wf}(T)dT + \int_{T_{IC}}^{T_{con}} C_p^{sorbent}(T)dT + \int_{T_{IC}}^{T_{con}} \rho_{liq}^{wf} \frac{(w_{max} + w_{min})}{2} C_p^{wf}(T)dT + Q_{sorption} \quad \textbf{(5)}$$

In the above equation, M_{w} is the molar mass of working fluid (water), ΔH_{ads} is the adsorption enthalpy, w_{max} and w_{min} are the maximal and minimum working capacity at sorption condition and desorption condition. T_{des} is the dessorption temperature. T_{IC} is the terminal temperature of the sorbent after Isosteric cooling. $C_{\mathrm{p}}^{\mathrm{sorbent}}$ and $C_{\mathrm{p}}^{\mathrm{wf}}$ are the specific heat capacity of sorbent (Supplementary Figure 19) and working fluid. Q_{sorption} is the energy released during adsorption of the sorbent, which can be calculated by:

$$Q_{sorption} = \frac{1}{M_w} \int_{w_{min}}^{w_{max}} \Delta H_{ads}(w)dw$$

$$Q_{regen} = \int_{T_{abs}}^{T_{des}} C_p^{sorbent}(T)\, dT + \int_{T_{abs}}^{T_{des}} \frac{w_{max}+w_{min}}{2} C_p^{wf}(T) - Q_{sorption} \quad \textbf{(6)}$$

Here, T_{des} and T_{abs} refers to the desorption temperature of sorbent and the temperature of absorption. $C_{\mathrm{p}}^{\mathrm{sorbent}}$ and $C_{\mathrm{p}}^{\mathrm{wf}}$ are the specific heat capacity of sorbent and working fluid.

where ΔH_{cond}(KJ Kg^{-1}) is the condensation enthalpy at the condenser temperature and $\Delta \mathrm{w}$ (KJ Kg^{-1}) is the working capacity of sorbent.

The average specific cooling power (SCP) can be calculated by the following equations: [2]

$$\mathrm{SCP} = \frac{0.8\Delta H_{eva}\,\Delta w}{\tau_{0.8ads}+\tau_{0.8des}} \quad \textbf{(7)}$$

where ΔH_{vap} (KJ Kg^{-1}) is the water enthalpy of evaporation, Δw is the working capacity of the LiCl@HGAFs-7/H_2O pair, $\tau_{0.8\mathrm{ads}}$ and $\tau_{0.8\mathrm{des}}$ are the adsorption and desorption times with the conversion q = 0.8 (Supplementary Figure 18, 20), which are 5368 s and 1726 s, respectively.

Supplementary Note 3. Measurement of Complex Permittivity.

To measure the microwave absorption performance of the composite fiber, the samples were cut into pieces and blended with paraffin (m_{fibers}:m_{paraffin}=3:7). Then the samples were pressed into concentric rings with an inner diameter of 3.04 mm and an out diameter of 7.00 mm. Using the coaxial method, based on the transmission line theory, a vector network analyzer was used to test the relative complex permittivity in the frequency range of 1-18 GHz. Since the sample is not magnetic, the complex permeability does not need to be considered.

Supplementary Note 4. Calculation details for Electromagnetic Absorption Performance from Complex Permittivity

The as-measured complex permittivity is used to calculate the electromagnetic (EM) absorption performance of materials with different thicknesses by the following equations:[5]

$$Z_{in} = Z_0\sqrt{\frac{\mu_r}{\varepsilon_r}}\,tanh\left[j\frac{2\pi}{c}\sqrt{\mu_r\varepsilon_r}fd\right] \quad \textbf{(8)}$$

$$\mathrm{RL} = 20\log\frac{|Z_{in}-Z_0|}{|Z_{in}+Z_0|} \quad \textbf{(9)}$$

where Z_{in} is the input impedance, Z_0 is the impedance of free space, the value is 367.77 Ω. c is the light velocity, f is the frequency, ε_r is the complex permittivity, and μ_r is the complex permeability.

The attenuation constant, α, determining the attenuation capability of the input EM wave can be indicated as:

$$\alpha = \frac{\sqrt{2}\pi f}{c} \times \sqrt{(\mu''\varepsilon'' - \mu'\varepsilon') + \sqrt{(\mu''\varepsilon'' - \mu'\varepsilon')^2 + (\mu''\varepsilon'' + \mu'\varepsilon')^2}} \quad \textbf{(10)}$$

where c is the light velocity, f is the frequency, ε' and ε'' is the real part and imaginary part of the complex permittivity, respectively.

Supplementary Note 5. Debye Relaxation

The polarization of dielectric materials is the phenomenon that bound charges to migrate along the direction of the applied electric field to produce an electron displacement or dipole orientation. In high-frequency electromagnetic fields, such as electron and dipole polarization relaxation, would be produced when the electron motion or dipole deflection cannot keep up with the change of alternating electric field. Based on Debye relaxation theory, the Cole-Cole semicircle equation can well make researchers understand the relaxation process that occurs under the reaction with alternating electromagnetic field:[6]

$$\varepsilon' = \varepsilon_\infty + \frac{\varepsilon_s - \varepsilon_\infty}{1+\omega^2\tau^2} \quad \textbf{(11)}$$

$$\varepsilon'' = \frac{\varepsilon_s - \varepsilon_\infty}{1+\omega^2\tau^2}\omega\tau + \frac{\sigma}{\omega\varepsilon_0} \quad \textbf{(12)}$$

Here, ε_∞ is the relative permittivity at a high-frequency limit, ε_s is the static permittivity, ω is the angular frequency, τ is the polarization relaxation time, and σ is the conductivity. From the equations above, the relationship between ε' and ε'' can be derived as follows:[6]

$$(\varepsilon' - \frac{\varepsilon_s + \varepsilon_\infty}{2})^2 + (\varepsilon'')^2 = (\frac{\varepsilon_s - \varepsilon_\infty}{2})^2 \quad \textbf{(13)}$$

In the plot, each Cole-Cole semicircle corresponds to a Debye relaxation process. If the absence of an obvious semicircle, it can be considered that the dielectric loss mainly comes from leakage conduction loss.

Supplementary Figures

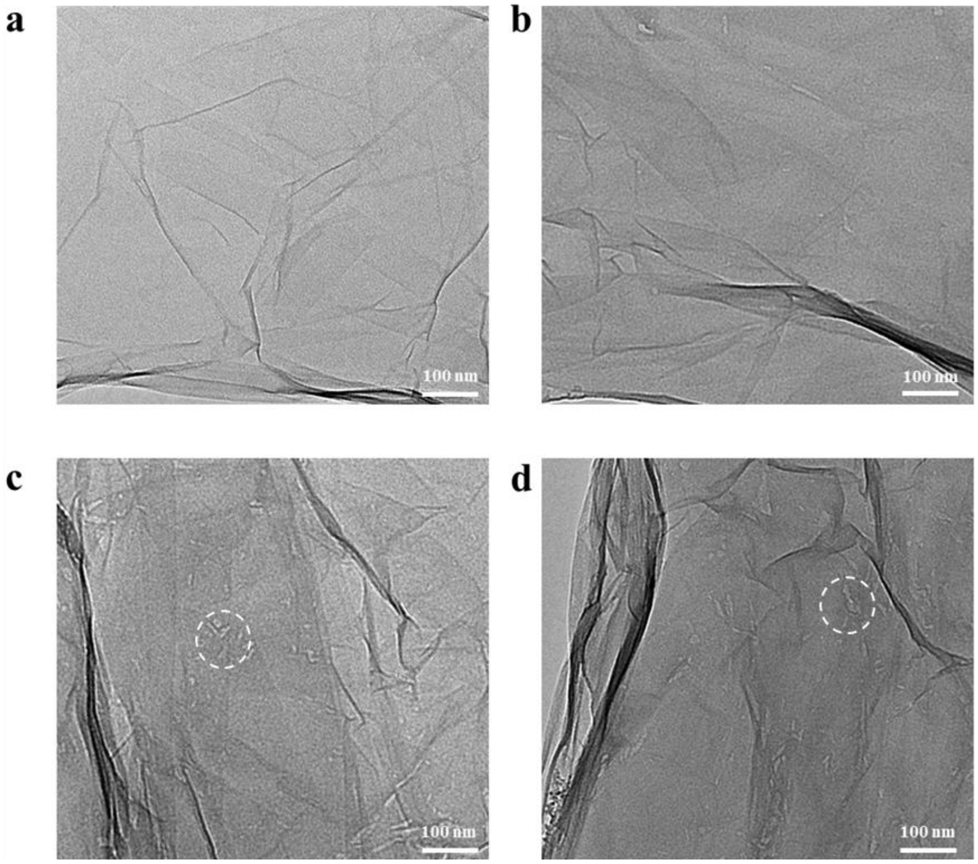

Supplementary Figure 1. TEM photo of a) GO and HGO made from etching GO by H_2O_2 for b) 0.5 h, c) 1 h and d)1.5 h.

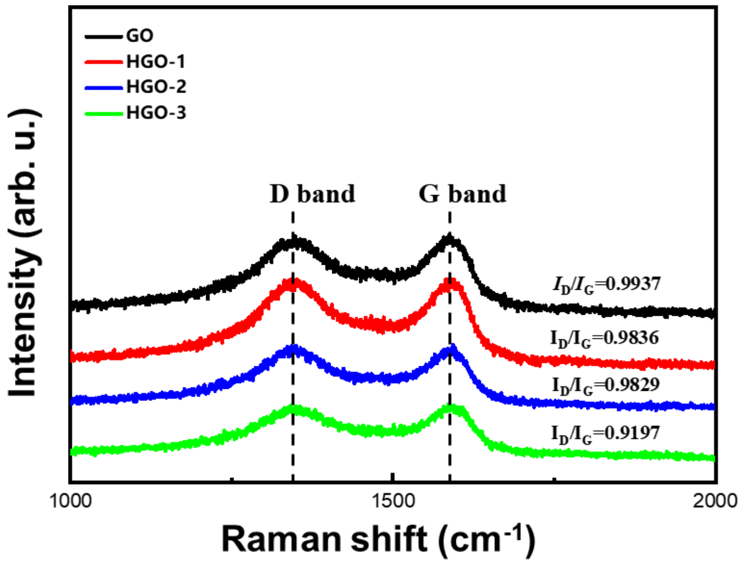

Supplementary Figure 2. Raman spectra of graphene oxide (GO) and holey graphene oxide (HGO) sheets. HGO-1, HGO-2, and HGO-3 correspond to H_2O_2

etching for 0.5 h, 1 h, and 1.5 h, respectively. It can be found that as the etching time increased, the intensity ratio decreased. The graphitic crystallite size (La) in the nanosheets can be estimated using the Tuinstra-Koenig equation ($L_a = 4.4\ I_g/I_d$).[7] The values for GO, HGO-1, HGO-2, and HGO-3 are 4.42 nm, 4.43 nm, 4.48 nm, and 4.78 nm.

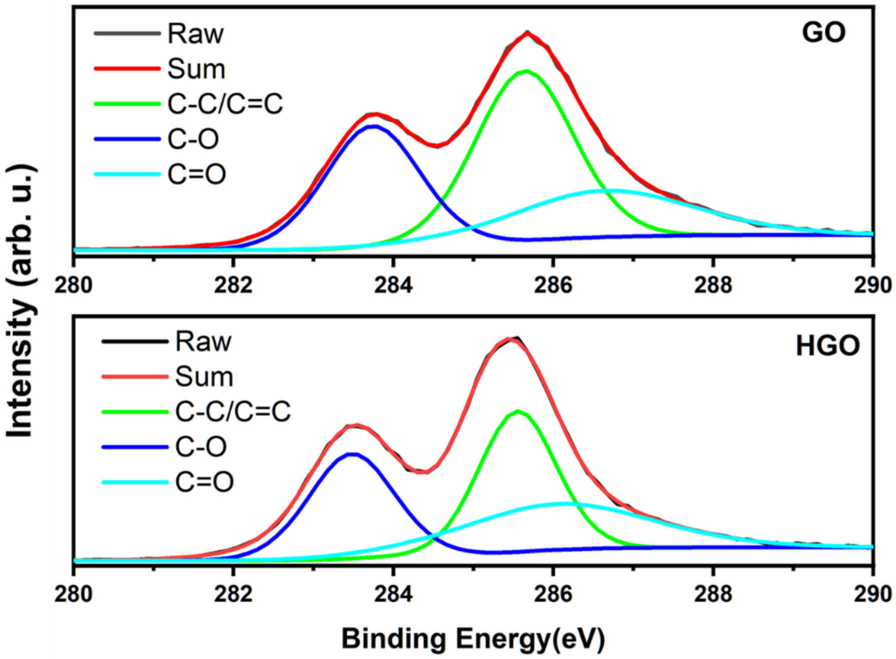

Supplementary Figure 3. C1*s* pattern for HGO-2 and GO, revealing HGO possesses fewer oxygen functional groups.

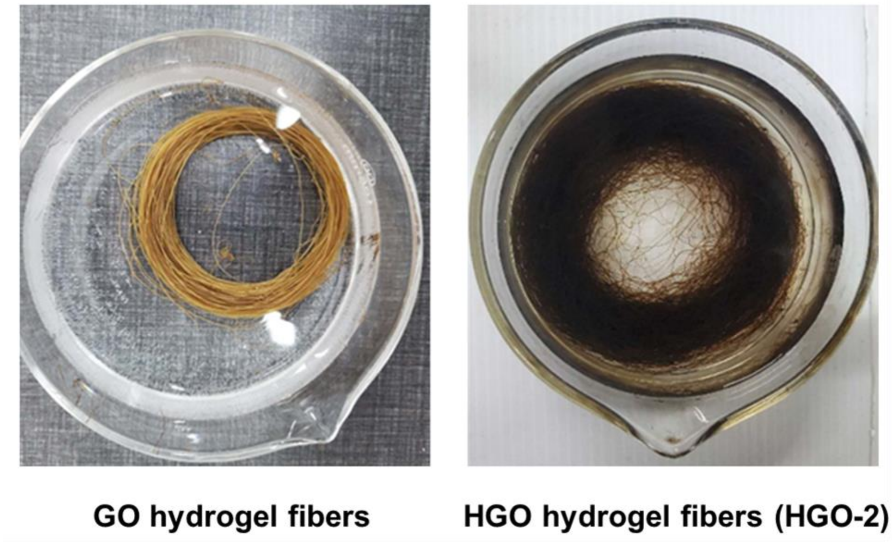

Supplementary Figure 4. A digital photo of GO hydrogel and HGO hydrogel

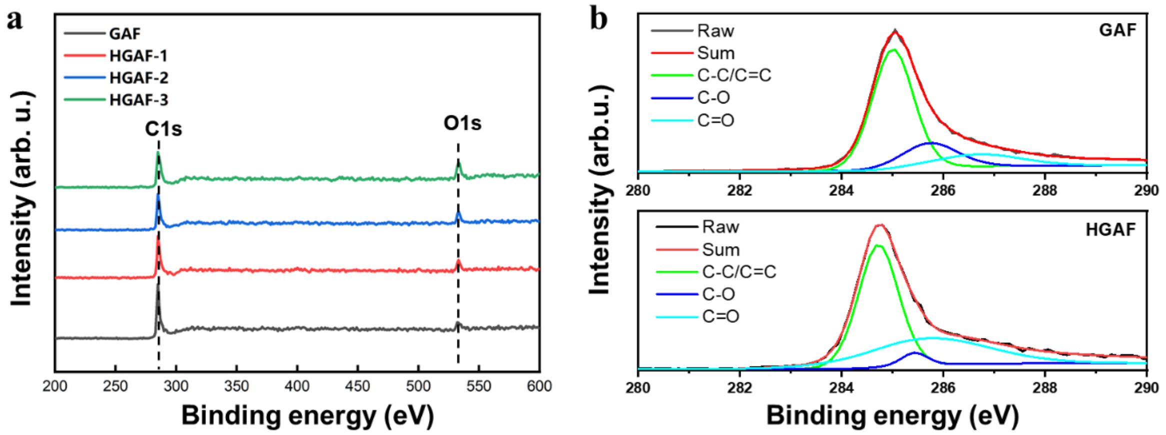

Supplementary Figure 5. a) XPS patterns of GAF, HGAF-1, HGAF-2, and HAGF-3 b) C1*s* patterns for GAF and HAGF-2

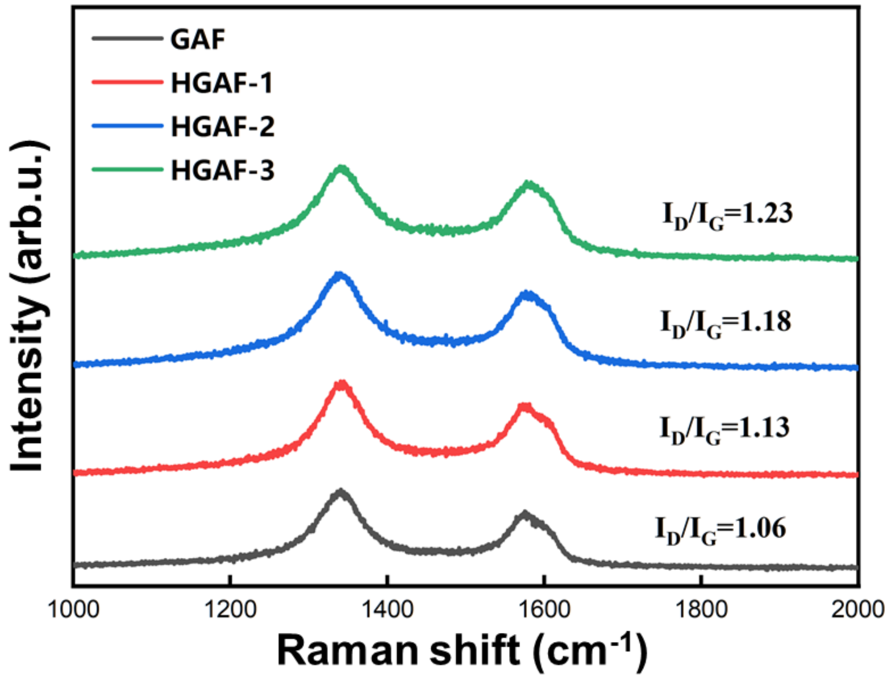

Supplementary Figure 6. Raman spectra of GAF, HGAF-1, HGAF-2, and HGAF-3.

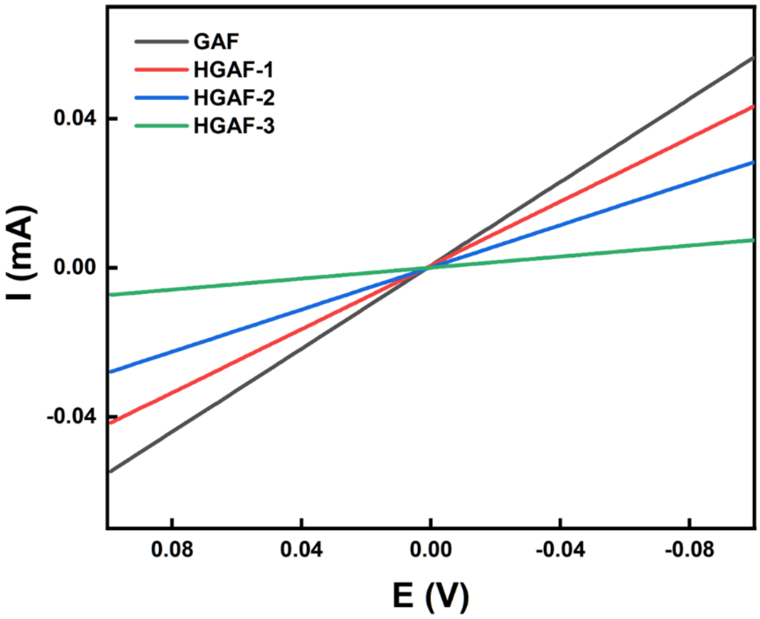

Supplementary Figure 7. I-E curves of GAF, HGAF-1, HGAF-2, and HGAF-3.

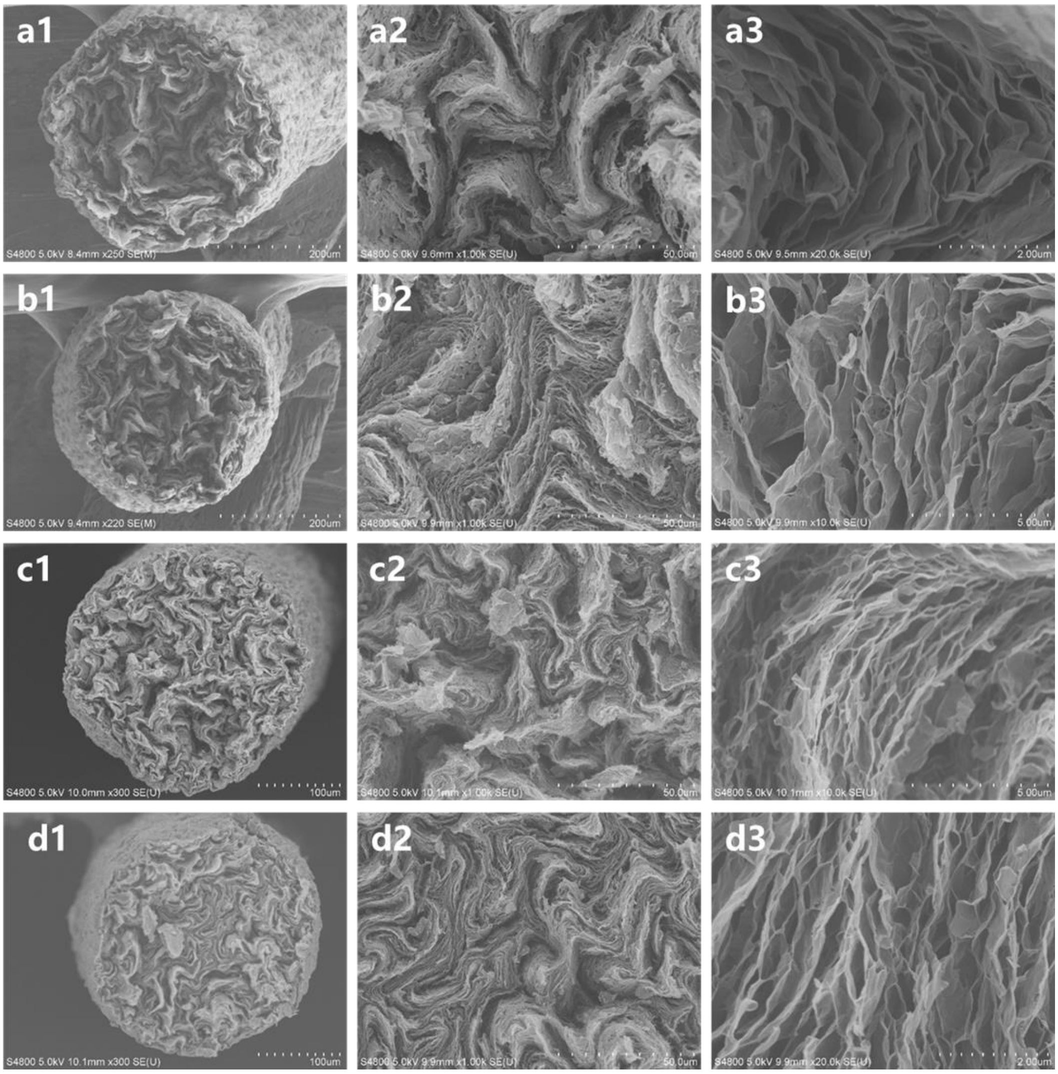

Supplementary Figure 8. SEM image of a) GAF, b) HGAF-1, c) HGAF-2, d) HGAF-3.

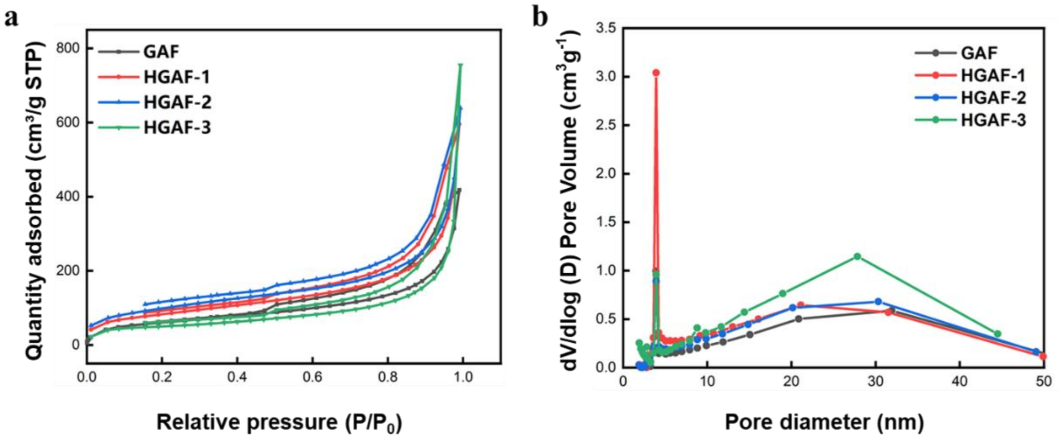

Supplementary Figure 9. a) N_2 sorption isotherms of GAF, HGAF-1, HGAF-2, and HAGF-3 b) pore size distribution GAF, HGAF-1, HGAF-2, and HAGF-3.

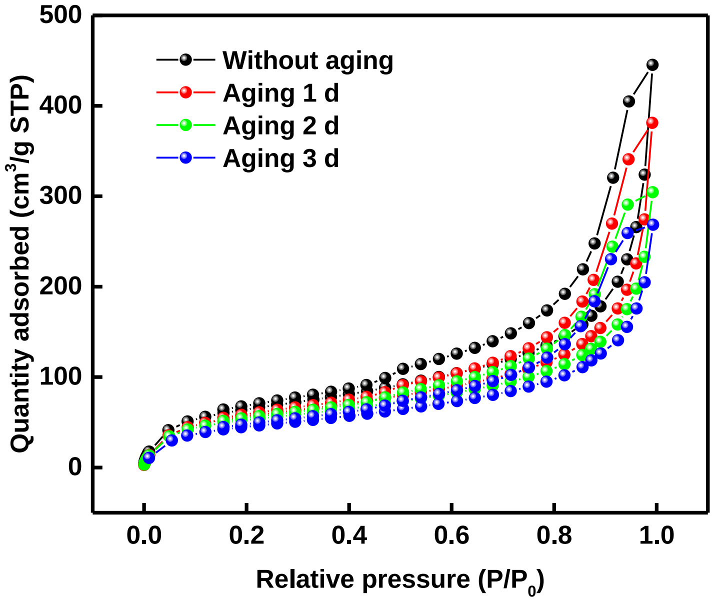

Supplementary Figure 10. N_2 sorption isotherms of HGAFs without aging and after aging for 1, 2, and 3 days.

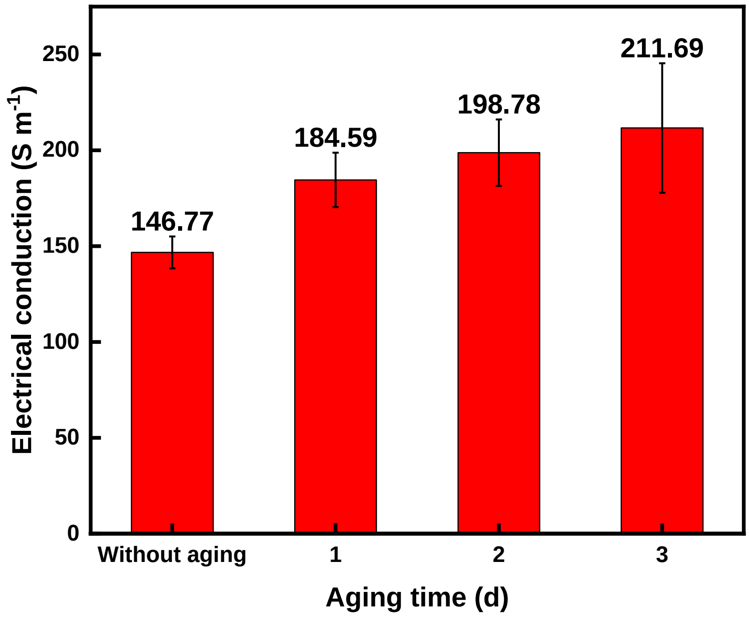

Supplementary Figure 11. Electrical conductivity of HGAFs without aging and after aging for 1, 2, and 3 days. Error bar represents the standard deviation, n = 5 in both panels (**a**) and (**b**).

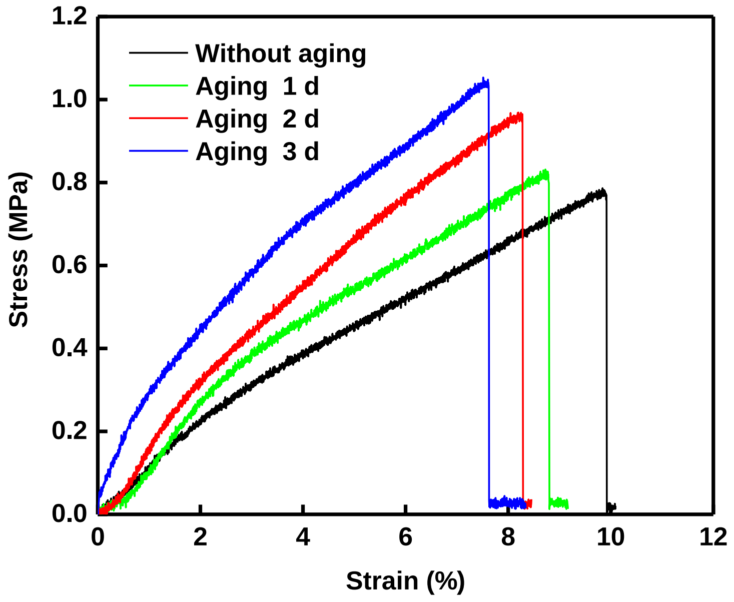

Supplementary Figure 12. Stress-strain curves of HGAFs without aging and after aging for 1, 2, and 3 days.

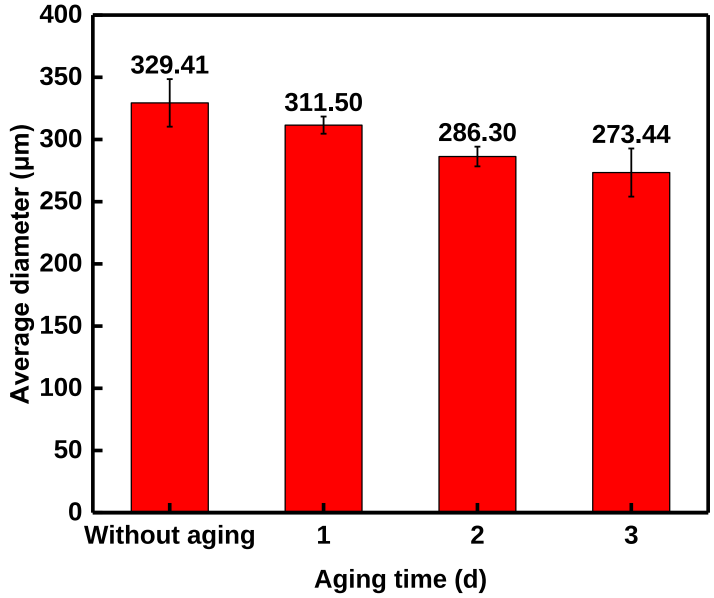

Supplementary Figure 13. The average diameter of HGAF without aging and after

aging for 1, 2, and 3 days. Error bar represents the standard deviation, n = 5 in both panels (**a**) and (**b**).

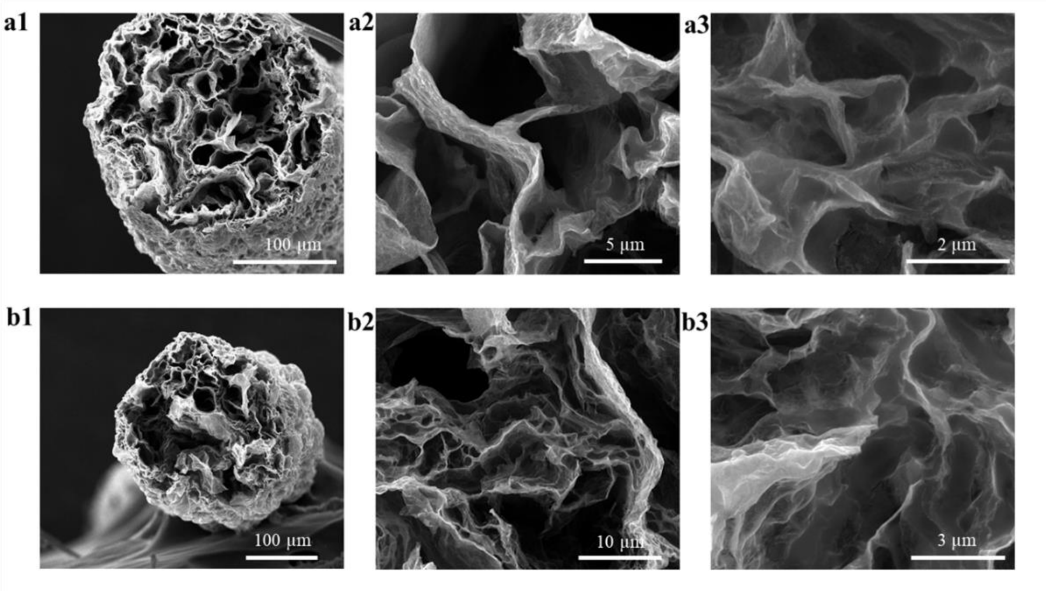

Supplementary Figure 14. SEM images of a) LiCl@HGAF-5 and b) LiCl@HGAF-7.

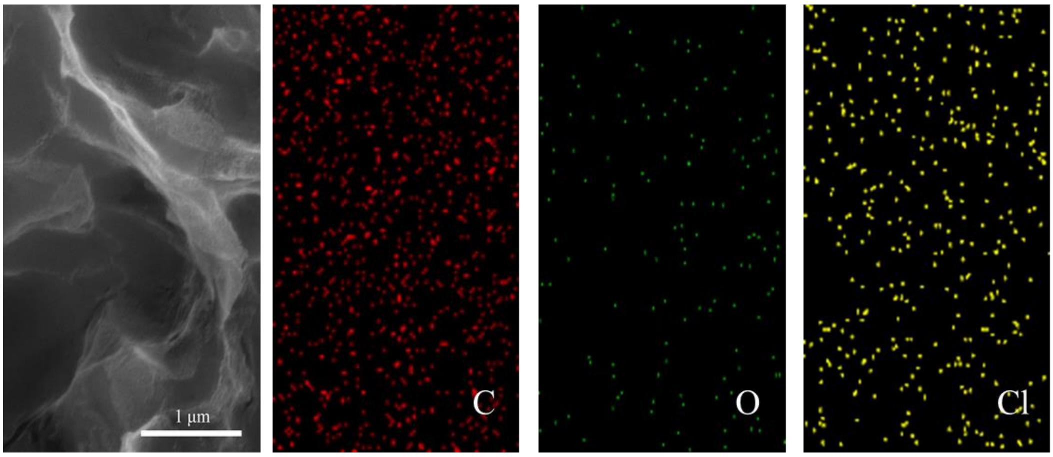

Supplementary Figure 15. SEM image of LiCl@HGAF-7 and corresponding element map.

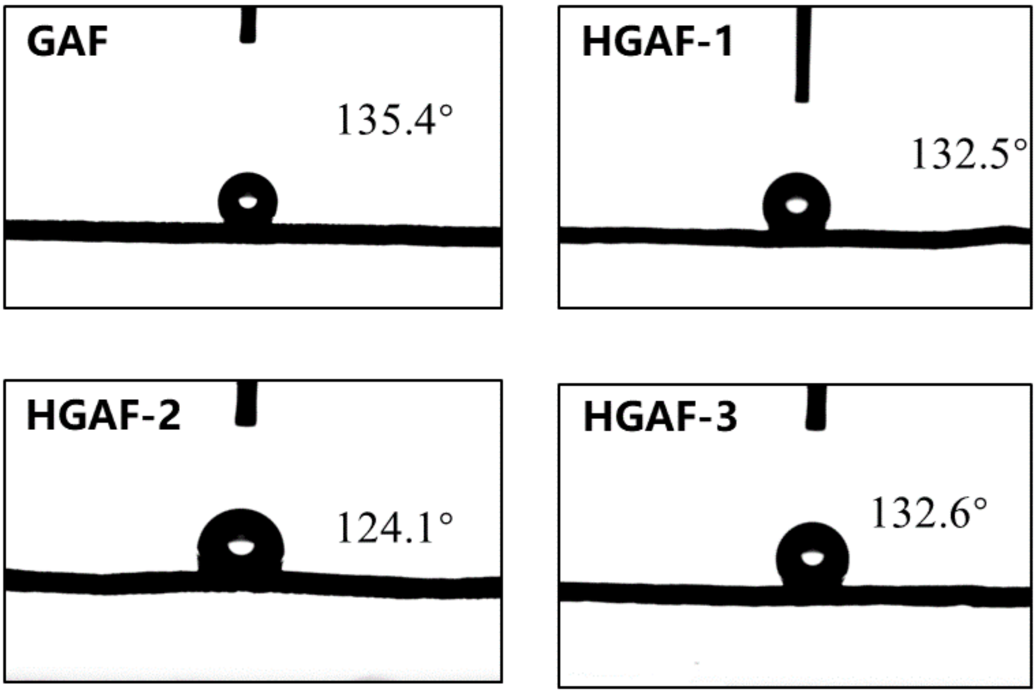

Supplementary Figure 16. Water contact angle with GAF, HAGF-1, HGAF-2, and HAGF-3.

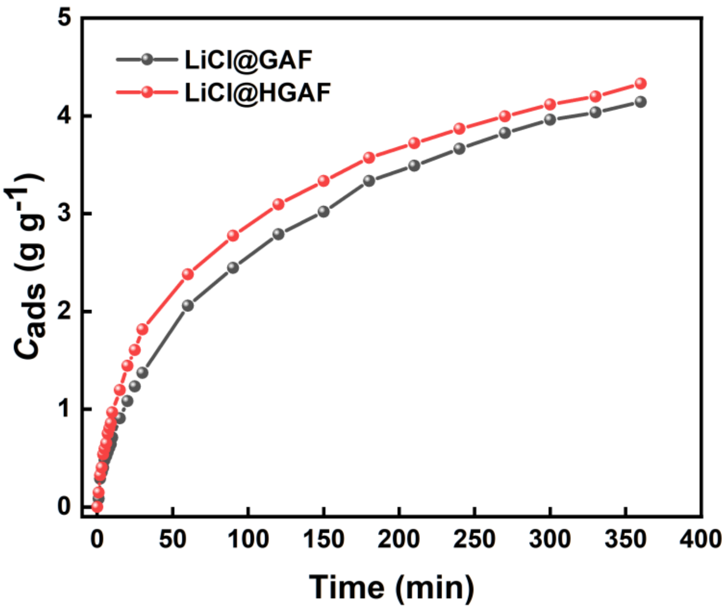

Supplementary Figure 17. The kinetic curves of LiCl@GAF-7 and LiCl@HGAF-7 at 90 RH%.

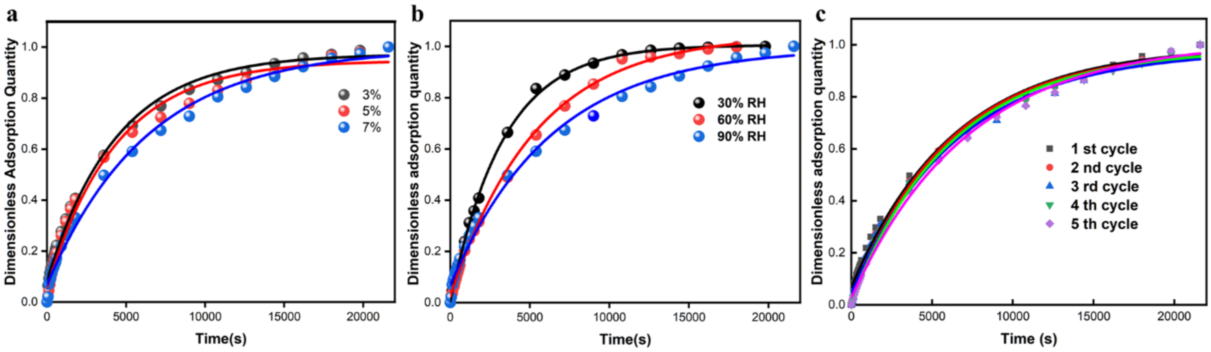

Supplementary Figure 18. a) Kinetic curves of LiCl@HGAF-7 with different salt content. b) Kinetic curves of LiCl@HGAF-7 at 30% RH, 60% RH, and 90% RH. c) Kinetic curves for 5 cycles.

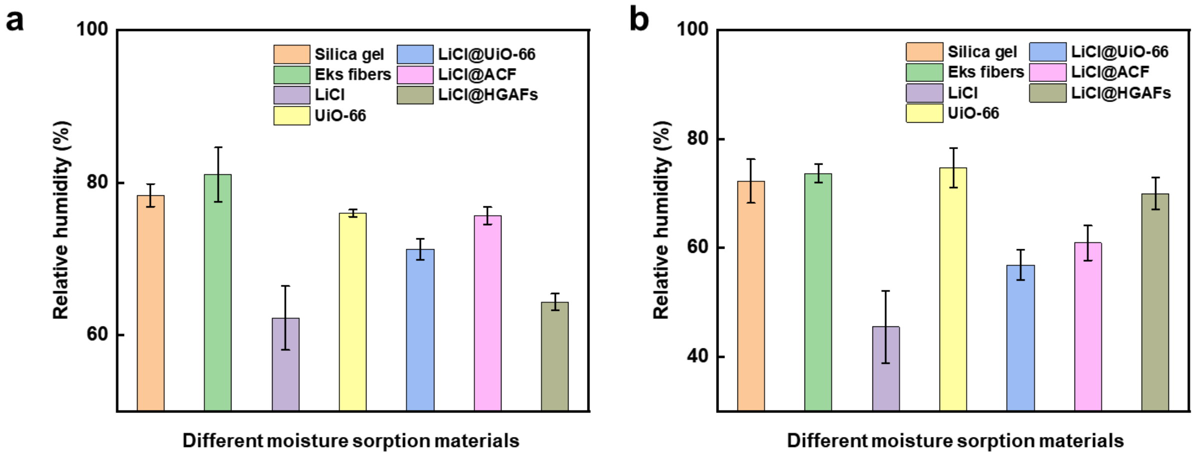

Supplementary Figure 19. Dehumidification performance of LiCl@HGAFs compared with other moisture sorption materials in a) the same mass (2 g) and b) the same packing volume (5 cm^3). The moisture sorption materials include commercial color-changing silica gel (methyl violet@silica gel), commercial hygroscopic fibers (EKS fibers from TOYOBO CO., LTD), LiCl, UiO-66 (zirconium 1,4-dicarboxybenzene MOF), LiCl@UiO-66, active carbon fiber loaded with lithium chloride (LiCl@ACF), and LiCl@HGAFs. The masses of moisture sorption materials with the same volume in b) are 3.55 g, 2.92 g, 2.74 g, 3.12 g, 2.84 g, and 1.76 g, respectively. Error bar represents the standard deviation, n = 3 in both panels (a) and (b).

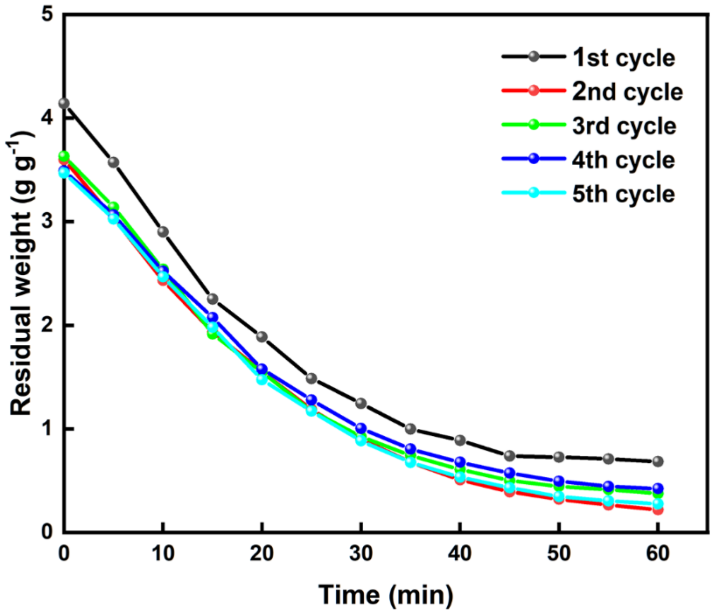

Supplementary Figure 20. Kinetic curves of LiCl@HGAF-7 for desorption at 5 cycles.

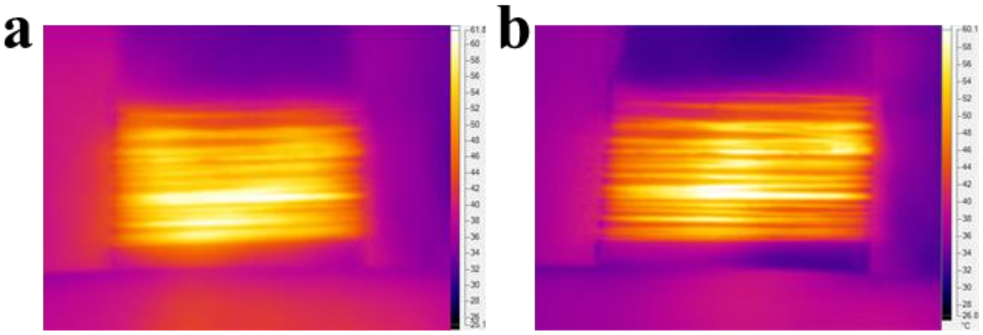

Supplementary Figure 21. IR images of a) HAGF b) LiCl@HGAF-7 under one sun irradiation.

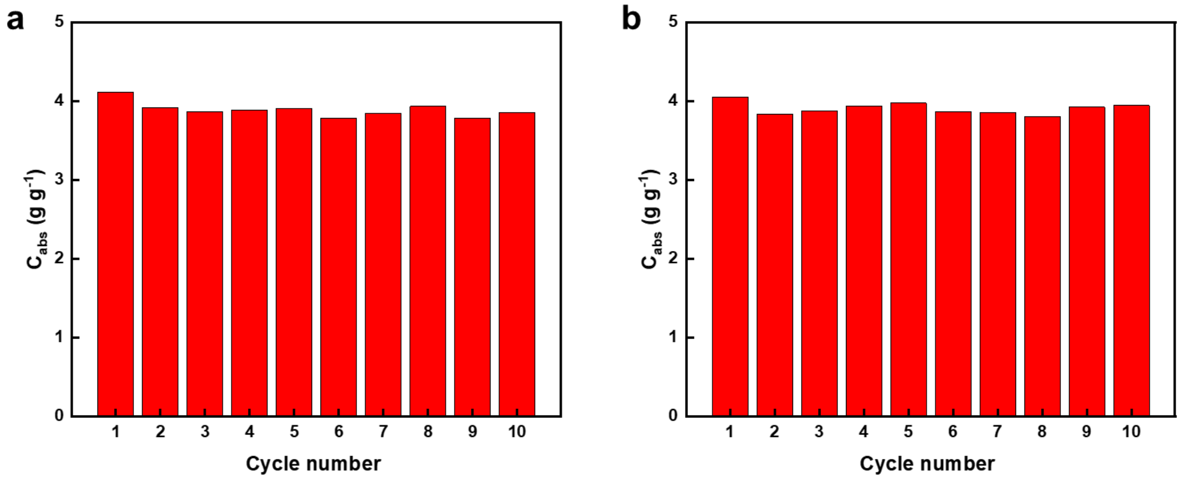

Supplementary Figure 22. Cycling stability of the sorption-desorption process of a)

LiCl@GAFs and b) LiCl@HGAFs.

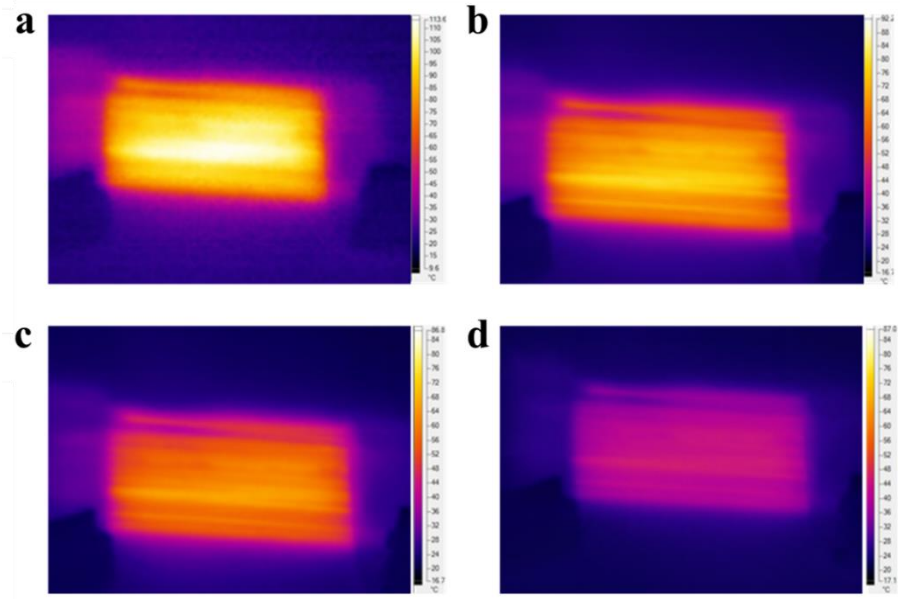

Supplementary Figure 23. IR images of LiCl@HGAF-7 under voltage of a) 12 V, b) 10 V, c) 8V and d) 6 V.

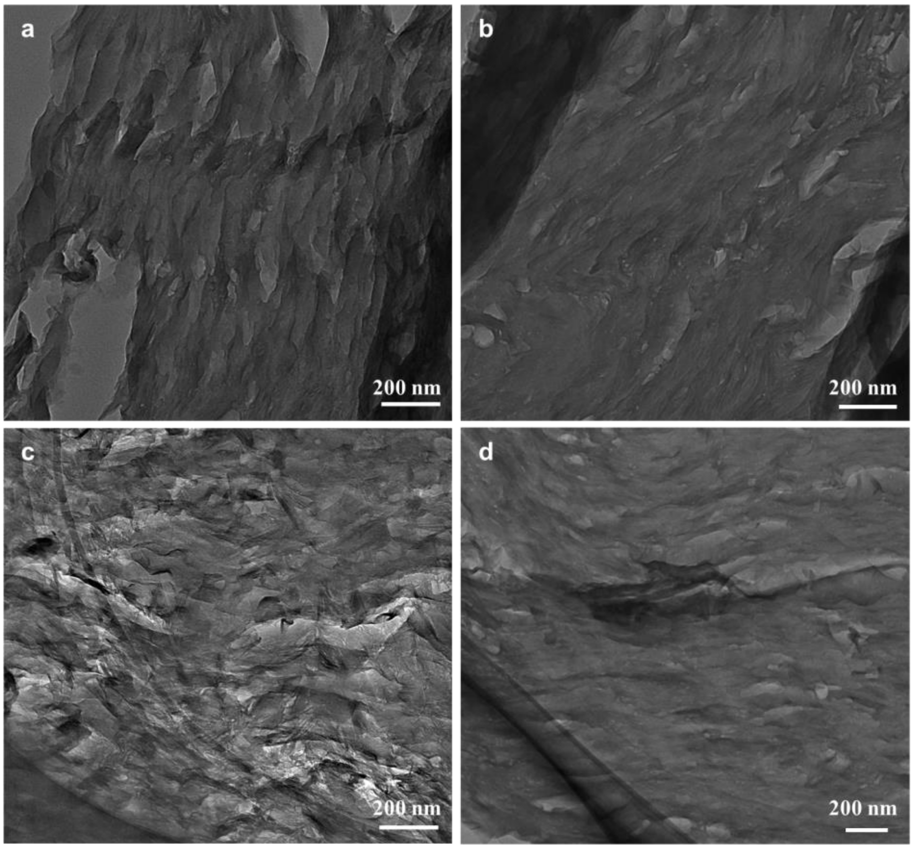

Supplementary Figure 24. TEM images of LiCl@GAF: a) before the cyclic test and b) after 10 sorption-desorption cycles, and TEM images of LiCl@HGAF: c) LiCl@HGAF before the cyclic test and d) after 10 sorption-desorption cycles.

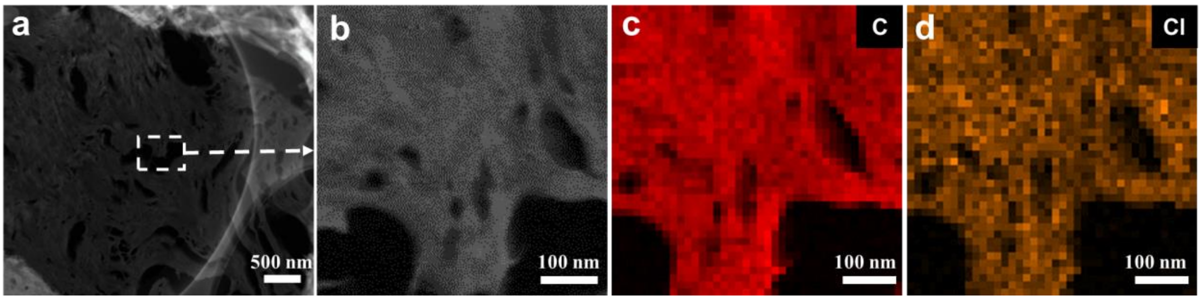

Supplementary Figure 25. TEM images (a, b) of LiCl@HGAF after 10 sorption-desorption cycles and the corresponding elemental maps: c) C element and d) Cl element.

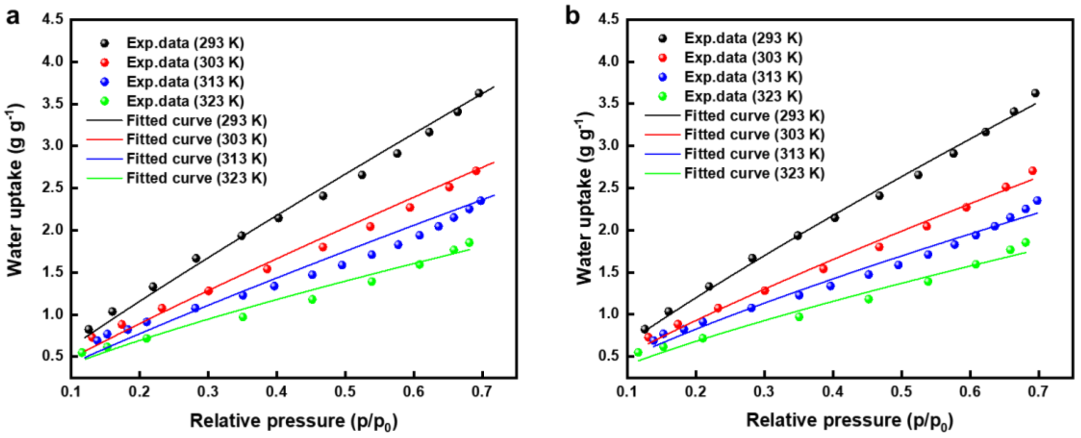

Supplementary Figure 26. Data fitting of the experimental water sorption isotherm of LiCl@HGAFs-7 by the a) Freundlich equation and b) S-B-K equation.

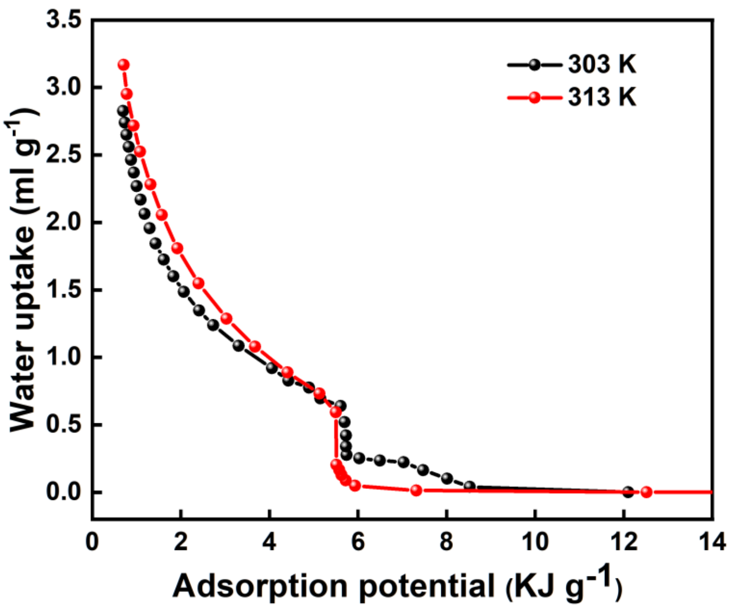

Supplementary Figure 27. Characteristic curves for water vapor adsorption in LiCl@HGAF calculated using isotherms at 303 K and 313 K. The characteristic curves are temperature invariant, justifying its usage to calculate isotherms at variant temperatures.

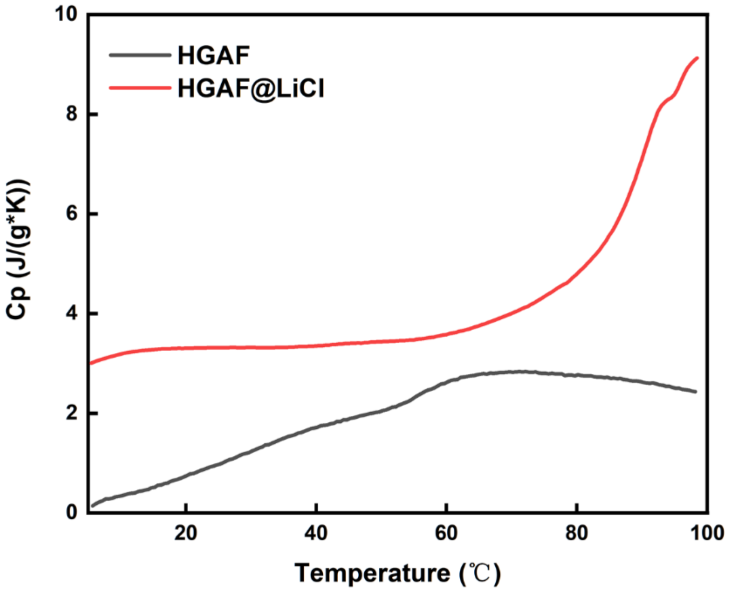

Supplementary Figure 28. The curves of specific heat capacity with temperature for HGAF and LiCl@HGAF-7.

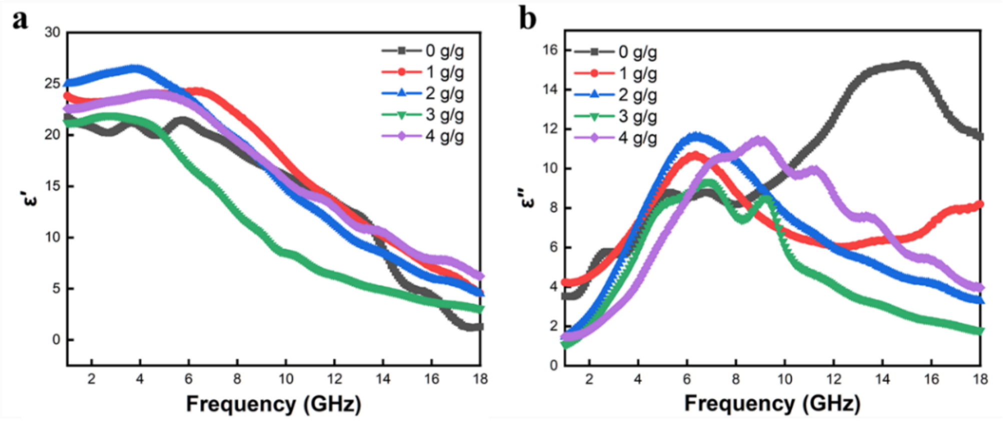

Supplementary Figure 29. Real permittivity and imaginary permittivity in 1-18 GHz for LiCl@HGAF and LiCl@HGAF-H_2O with the water content of 1 g g^{-1}, 2 g g^{-1}, 3 g g^{-1}, and 4 g g^{-1}.

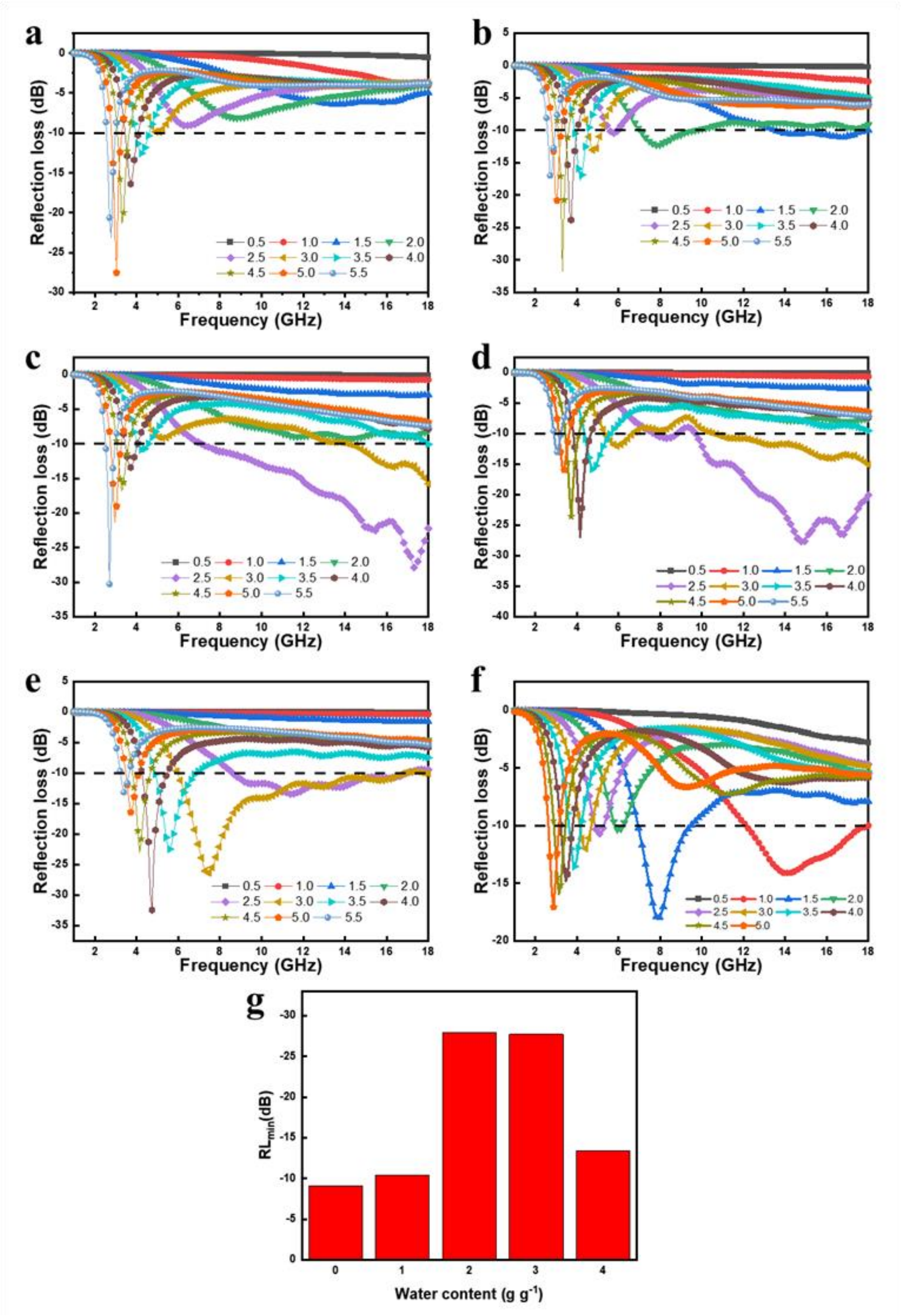

Supplementary Figure 30. Reflection loss of LiCl@HGAF-7-H_2O with different water contents and HGAF at different thicknesses. a) 0 g g^{-1}, b) 1 g g^{-1}, c) 2 g g^{-1}, d) 3 g g^{-1}, e) 4 g g^{-1}, and f) HGAF g) Minimum reflection loss of LiCl@HGAF-7-H_2O with different water contents at the thickness of 2.5 mm.

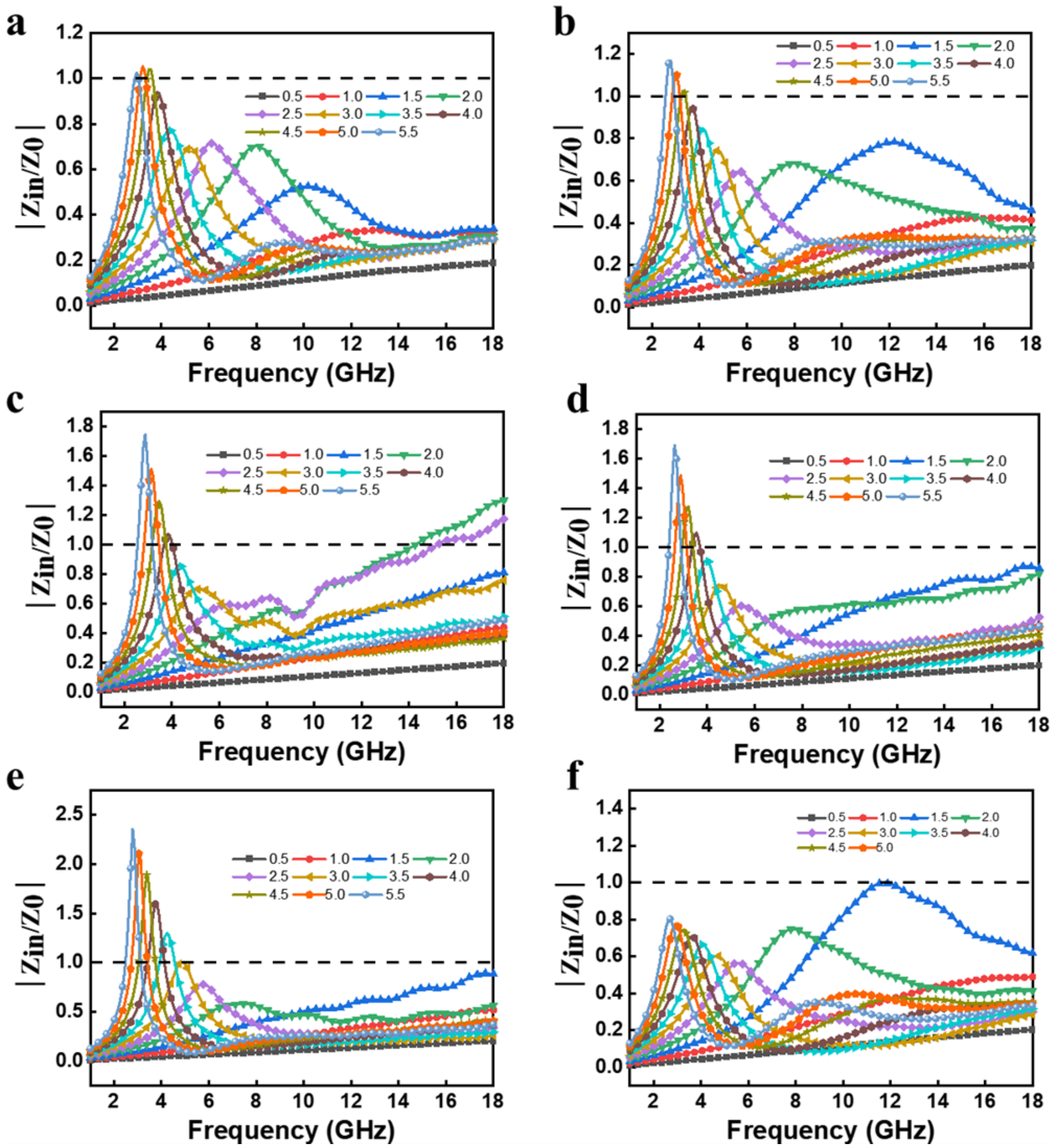

Supplementary Figure 31. $|Z_{in}/Z_0|$ of LiCl@HGAF-7-H_2O with different water content and HGAF at different thicknesses. a) 0 g g^{-1}, b) 1 g g^{-1}, c) 2 g g^{-1}, d) 3 g g^{-1}, e) 4 g g^{-1}, and f) HAGF.

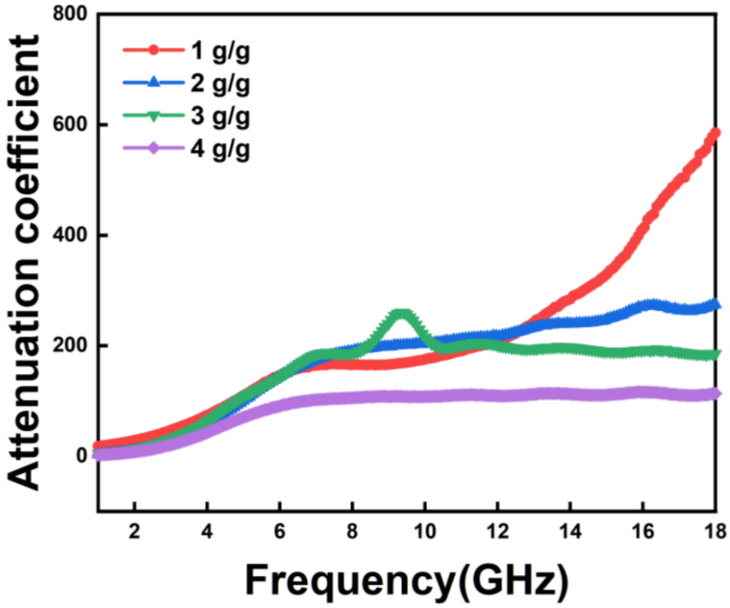

Supplementary Figure 32. Attenuation constant of LiCl@HGAF with different water contents.

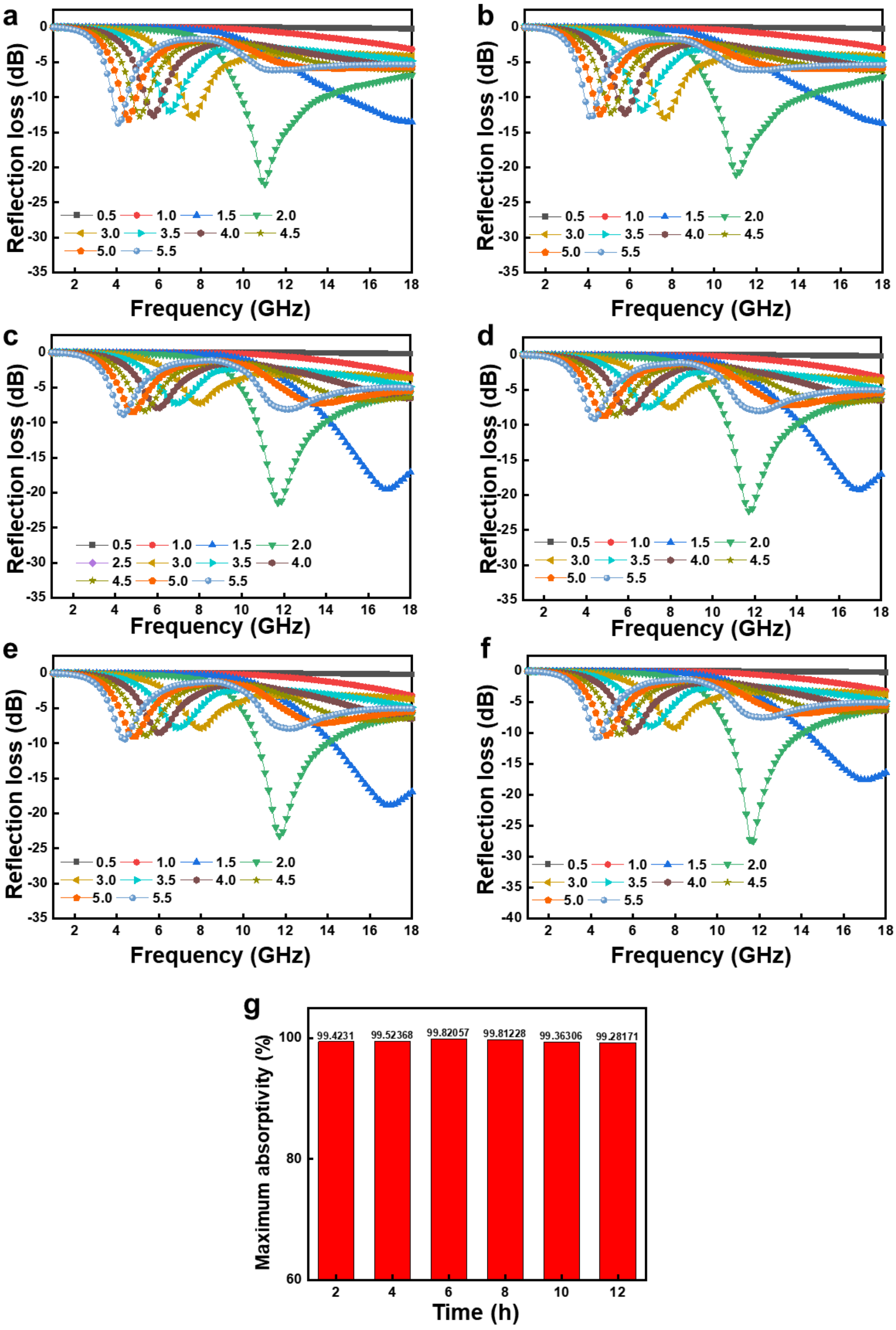

Supplementary Figure 33. The sorption stability of microwave test for LiCl@HGAF-H_2O at different thicknesses. The samples were placed in the electromagnetic environment for a) 2, b) 4, c) 6, d) 8, e) 10 and f) 12 h, respectively. g) Maximum absorptivity (%) with the thickness of 2.0 mm with different radiation times.

Supplementary Tables

Supplementary Table 1 Kinetic parameters from gravimetric water sorption over time, obtained from fitting the gravimetric measurement with equation (1).

Sample	RH[%]	Kinetic equations	K_s [10^{-4} s^{-1}]	R^2
**LiCl@HGAF-7**	**30**	$y = 1 - e^{2.998*10^{-4}t}$	**2.998**	**0.99966**
**LiCl@HGAF-7**	**60**	$y = 1 - e^{1.855*10^{-4}t}$	**1.855**	**0.99822**
**LiCl@HGAF-7**	**90**	$y = 1 - e^{1.653*10^{-4}t}$	**1.653**	**0.99378**
**LiCl@HGAF-5**	**90**	$y = 1 - e^{2.290*10^{-4}t}$	**2.290**	**0.99568**
**LiCl@HGAF-3**	**90**	$y = 1 - e^{2.388*10^{-4}t}$	**2.388**	**0.99336**
**LiCl@GAF-7**	**90**	$y = 1 - e^{1.566*10^{-4}t}$	**1.566**	**0.99778**
**LiCl@HGAF-7-2[i)]**	**90**	$y = 1 - e^{1.600*10^{-4}t}$	**1.600**	**0.99748**
**LiCl@HGAF-7-3[i)]**	**90**	$y = 1 - e^{1.575*10^{-4}t}$	**1.575**	**0.99028**
**LiCl@HGAF-7-4[i)]**	**90**	$y = 1 - e^{1.533*10^{-4}t}$	**1.533**	**0.99422**
**LiCl@HGAF-7-5[i)]**	**90**	$y = 1 - e^{1.393*10^{-4}t}$	**1.393**	**0.99326**

[i)]The sample named LiCl@HGAF-7-x means that the sample LiCl@HGAF-7 through x cycles.

Supplementary Table 2. Coefficients of the Freundlich equation for LiCl@HGAFs-7

	293 K	303 K	313 K	323 K
X_0	5.013	3.783	3.249	2.842
n	1.099	1.116	1.122	1.226

Supplementary Table 3. Coefficients of the S-B-K equation for LiCl@HGAFs.

Parameters	Value
A0	$3.1119*10^{3}$
A1	-29.657
A2	$9.4463*10^{-2}$
A3	$-1.0047*10^{-4}$
B0	$-2.0222*10^{2}$
B1	1.9929
B2	$-6.5048*10^{-3}$
B3	$7.06*10^{-6}$

Supplementary References

1. Sun, Y. et al. Tunable LiCl@UiO-66 composites for water sorption-based heat transformation applications. *J. Mater. Chem. A* **8**, 13364-13375 (2020).

2. Garzón-Tovar, L. et al. Composite salt in porous metal-organic frameworks for adsorption heat transformation. *Adv. Funct. Mater.* **27**, 1606424 (2017).

3. Cho, K. H. et al. Rational design of a robust aluminum metal-organic framework for multi-purpose water-sorption-driven heat allocations. *Nat. Commun.* **11**, 5112 (2020).

4. de Lange, M. F. et al. Adsorption-driven heat pumps: the potential of metal-organic frameworks. *Chem. Rev.* **115**, 12205 (2015).

5. Che, R. C. et al. Microwave absorption enhancement and complex permittivity and permeability of Fe encapsulated within carbon nanotubes. *Adv. Mater.* **16**, 401-405 (2004).

6. Lu, A. et al. Wearable aramid–ceramic aerogel composite for harsh environment. *Adv. Eng. Mater.* **23**, 2001169 (2021).

7. Cançado, L. G. et al. General equation for the determination of the crystallite size La of nanographite by Raman spectroscopy. *Appl. Phys. Lett.* **88**, 163106 (2006).
